# Characteristics and structural imbalances of biomedical academicians in China: policy implications for the governance of leading medical talents

**DOI:** 10.3389/fpubh.2026.1944514

**Published:** 2026-10-23

**Authors:** Rong Liu, De Fei Huang, Tao Huang, Tian Lan Hu

**Affiliations:** 1 Department of Ultrasound Medicine, Tianyou Hospital Affiliated to Wuhan University of Science and Technology, Wuhan, China; 2 Management School, Sheffield University, Sheffield, United Kingdom; 3 School of Law and Economics, Wuhan University of Science and Technology, Wuhan, China; 4 Health Examination Center, Tianyou Hospital Affiliated to Wuhan University of Science and Technology, Wuhan, China

**Keywords:** biomedical academicians, governance of medical talents, human resources for health, leading medical talents, structural imbalance

## Abstract

**Background:**

Against the backdrop of intense global competition in biomedical science, leading biomedical talents constitute core determinants of national strategic science and technology capacity and effective health workforce governance. Nevertheless, large-sample empirical research on career trajectories and structural disparities among China’s elite biomedical academics remains limited, constraining evidence-based talent policy refinement.

**Methods:**

This retrospective study included 284 biomedical academicians listed on the official websites of the Chinese Academy of Sciences (CAS) and the Chinese Academy of Engineering (CAE) as of July 1, 2025. Drawing on publicly available data on demographics, educational background, mentorship, and institutional affiliations, it systematically characterized the distribution of top-tier talent and the factors influencing age at election, using descriptive statistics, hypothesis testing, and multiple linear regression analysis.

**Results:**

The average age at election for CAS biomedical academicians was significantly lower than that for CAE academicians (*p* < 0.001). Female academicians constituted only 12.68% (36/284) of the total sample. A significant geographical clustering was observed in the birthplaces and upbringing regions of the academicians. Univariate analysis showed that academicians holding overseas doctoral degrees or supervised by academicians were elected at younger ages. However, after adjustment for confounders in multiple linear regression, overseas doctoral degree was not independently associated with age at election (β = −0.109, *p* = 0.071). In contrast, highest degree attainment and supervised by academicians remained statistically significant predictors. Over half of the academicians were employed by universities, revealing an imbalance in the ratio of Basic Medical Sciences to Clinical Medicine talent. The educational attainment of academicians in the <50 and 50–65 age groups was significantly higher than that of those over 65 (*p* < 0.05).

**Conclusion:**

Chinese biomedical academician cohort exhibits substantial structural imbalances across gender, geography, age, and academic career trajectories. Drawing on the WHO global health workforce governance framework, this study proposes governance insights across three dimensions: national top-level policy design, institutional platform development, and early-career talent cultivation. These findings may inform future policy research and empirical evaluation.

## 1 Introduction

The global health system faces the dual challenges of a chronic shortage and inequitable distribution of high-level health workers. The World Health Organization (WHO) projects a global shortfall of 11 million health professionals by 2030 (1, 2). Against the backdrop of national strategies such as “Healthy China” and “Rejuvenating the Country through Science, Education, and Human Resources”, cultivating high-end biomedical talent with original scientific research leadership is key to enhancing the core competitiveness of the national biomedical industry.

Biomedical academicians mainly refer to members of the CAS Division of Life Sciences and Medical Sciences and the CAE Department of Medicine and Health. They represent the highest tier of academic talent in China across biomedical sciences, clinical medicine, public health, pharmacy, and life sciences. Tasked with generating original research breakthroughs, developing key technologies, building disciplinary systems, and nurturing subsequent talent cohorts, this group constitutes China’s top academic leadership in biomedicine. Leading medical talents are outstanding experts and academic leaders in biomedicine with strong professional competence, forward-looking vision, and broad academic influence. As the backbone of disciplinary development, clinical technology advancement, research innovation, and team building, they form an intermediate talent pipeline connecting young medical researchers and academicians.

Structural imbalance in this paper refers to skewed developmental patterns among top medical talents across gender, geography, discipline, and access to training resources. Such disparities emerge from interactions between talent evaluation systems, resource allocation models, training mechanisms, and policy priorities. These imbalances are asymmetric and self-reinforcing; they cannot be spontaneously corrected within academic systems. Over time, they widen gaps in talent development, reduce biomedical innovation efficiency, and hinder balanced progress in health services. The talent governance framework adopted here has four core dimensions. Equity means universal access to talent development resources and opportunities. Balance describes the rational distribution of talents across regions, disciplines, and gender groups. Sustainability refers to long-term continuity in talent succession and supportive growth ecosystems. Inclusiveness and diversity represent a talent development environment that removes barriers related to identity, gender, and geography. The four dimensions are interrelated yet have distinct priorities.

Current domestic research on medical talent has certain limitations: existing studies predominantly focus on micro-level teaching reforms such as curricula and clinical training (3, 4), lacking large-sample quantitative analyses of the entire cohort of top-tier, national academician-level talent (5). A small body of academician studies only describes single dimensions including disciplinary profiles, gender differences, ethnic composition, and geographic mobility (6–9). International literature has more commonly examined structural barriers to gender equity among leading medical talents (10, 11) and regional health workforce shortages (12). Studies of talent growth drivers have centered on individual motivation, education systems, policy support, and social contexts (13, 14). Research on cultivating leading medical talents covers four major themes: international collaborative training, gender equity, leadership development, and mentorship systems (15–18). Taken together, existing research mostly covers academicians across all disciplines or talents in the broad medical field, yet specialized studies focusing on academicians from the Chinese Academy of Sciences and the Chinese Academy of Engineering in the biomedical field remain scarce. Moreover, few studies simultaneously integrate multidimensional information such as demographics, educational trajectories, mentorship, supporting institutions, and disciplinary distribution. Most previous studies have only employed univariate comparisons, making it difficult to distinguish the independent effects of various factors under conditions of mutual confounding, and cannot distinguish the independent contributions of mentorship and overseas educational experiences during the critical phase of talent development for top-tier talents. This study adopts a data-driven approach to analyze the career trajectories and shared characteristics of biomedical academicians, providing practical evidence to reform existing talent cultivation models.

The WHO 2026 National Health Workforce Accounts Report notes that persistent inequities and imbalances in the geographical distribution, demographic structure, and skill mix of the health workforce will continue to hinder the achievement of universal health goals, becoming a key bottleneck constraining biomedical innovation and sustainable development (1). The formation, growth, and sustained supply of China’s biomedical academician cohort are not only vital to national scientific and technological competitiveness but also strategically relevant to balanced development of the global biomedical innovation ecosystem. Given these deep-rooted structural challenges, optimizing training pathways for high-end biomedical talents is both necessary and urgent (19). This study uses a cross-sectional observational design combined with retrospective data analysis. It conducts multidimensional quantitative analysis of the current CAS and CAE biomedical academician cohorts, systematically identifies structural imbalances, interprets findings from a health workforce governance perspective, and proposes policy recommendations at national, institutional, and individual levels.

## 2 Data sources and research methods

### 2.1 Study sample and data sources

The sample consisted of 284 biomedical academicians listed on the official websites of CAS and CAE on July 1, 2025, including 153 members from the CAS Division of Life Sciences and Medical Sciences and 131 members from the CAE Department of Medicine and Health. All data were retrieved from public official platforms of CAS, CAE, and their affiliated universities and hospitals. Collected indicators covered four dimensions with standardized operational definitions: (1) Demographic characteristics: gender, birth year, birthplace, and age at election (calculated from the official election year announced by the two academies); (2) Educational background: undergraduate, master’s, and doctoral alma maters; highest academic degree; overseas study experience (formal bachelor’s or higher education at foreign institutions, excluding short-term visits or exchanges); overseas doctoral degree (doctoral degree obtained from institutions outside mainland China); (3) Academician supervisor: whether the scholar was supervised by an academician during master’s or doctoral training, excluding later academic collaboration or short-term advisory relationships; (4) Employer and disciplinary characteristics: primary affiliation and core research discipline.

Operational definitions of key variables are listed below: ① Age at election: continuous variable calculated as the official election year minus birth year. ② Gender: male, female. ③ Prestige of undergraduate institution: whether the undergraduate institution belonged to Project 985 or Project 211 (historic national initiatives for elite university development in China), an overseas institution (classified as prestigious), or a regular domestic institution. ④ Highest degree: categorized as doctoral, master’s, or bachelor’s degree. ⑤ Overseas doctoral degree: binary indicator for whether the scholar obtained a doctoral degree outside mainland China. ⑥ Academician supervisor: binary indicator for whether the master’s or doctoral supervisor was a CAS or CAE academician. ⑦ Age group at election: young (<50 years), middle-aged (50–65 years), older (>65 years). ⑧ Type of primary employing institution: research institute (including CAS-affiliated institutes), university, hospital, government agency, public health institution or academic society, enterprise, or other.

Data processing followed standard academic protocols. Two researchers independently extracted indicators from the academies’ official websites and public resumes of affiliated universities and hospitals, then cross-checked records. Missing information was supplemented through public news reports, individual academic resumes, and university talent recruitment materials, resulting in a final dataset with 100% completeness. Data were anonymized to protect privacy: personally identifiable information (names, specific work departments) was removed, retaining only aggregated statistical variables.

In accordance with the *Ethical Review Measures for Life Sciences and Medical Research Involving Humans* (Guowei Kejiao Fa [2023] No. 4) issued by the National Health Commission of China, ethics review may be exempted for research using: (1) lawfully obtained public data or observational data collected without interfering with human subjects; or (2) anonymized data, provided the research causes no harm to individuals and does not involve sensitive personal information or commercial interests. This study meets these exemption criteria: it used lawfully obtained anonymized public data, involved no human subject intervention, and contained no sensitive personal information or commercial interests. Therefore, institutional ethics committee review and informed consent were not required.

### 2.2 Statistical analysis

Data processing and statistical analysis were performed using SPSS 22.0 software. Categorical variables were described using counts and percentages, with group comparisons made using the chi-square test. Continuous variables were expressed as mean ± SD. The Shapiro–Wilk test was used to assess normality, and Levene’s test for homogeneity of variances. Comparisons between two groups for normally distributed continuous variables used the independent samples *t*-test, while non-normally distributed data were analyzed using the Mann–Whitney U test. Comparisons among multiple groups used one-way ANOVA and the Kruskal–Wallis H test. We constructed a multiple linear regression model, with the age at election as the dependent variable, and six independent predictors: gender, prestige of undergraduate institution, highest academic degree, overseas doctoral degree, background of academician supervisor, and type of employer. The variance inflation factor (VIF) and condition index were used to evaluate multicollinearity. Cook’s distance assessed model robustness. A *p*-value < 0.05 was considered statistically significant.

## 3 Results

### 3.1 Overall distribution of election age for academicians of both academies

The average age at election for the 284 biomedical academicians was 57.63 ± 7.18 years, ranging from 39 to 76 years. The youngest elected academician from the CAS was 39 years old, with 29 academicians elected under the age of 50. The youngest from the CAE was 41 years old, with only 8 elected under the age of 50.

### 3.2 Gender composition and election trends over time

A total of 36 female academicians were identified, constituting 12.68% (36/284) of the total sample, with 12.41% (19/153) from the CAS and 12.97% (17/131) from the CAE. Among the 103 newly elected academicians from 2015 to 2024, only 12.62% (13/103) were female. The peak annual number of newly elected female academicians was 4, with three election cycles having no female candidates elected (Figure 1).

**Figure 1:**
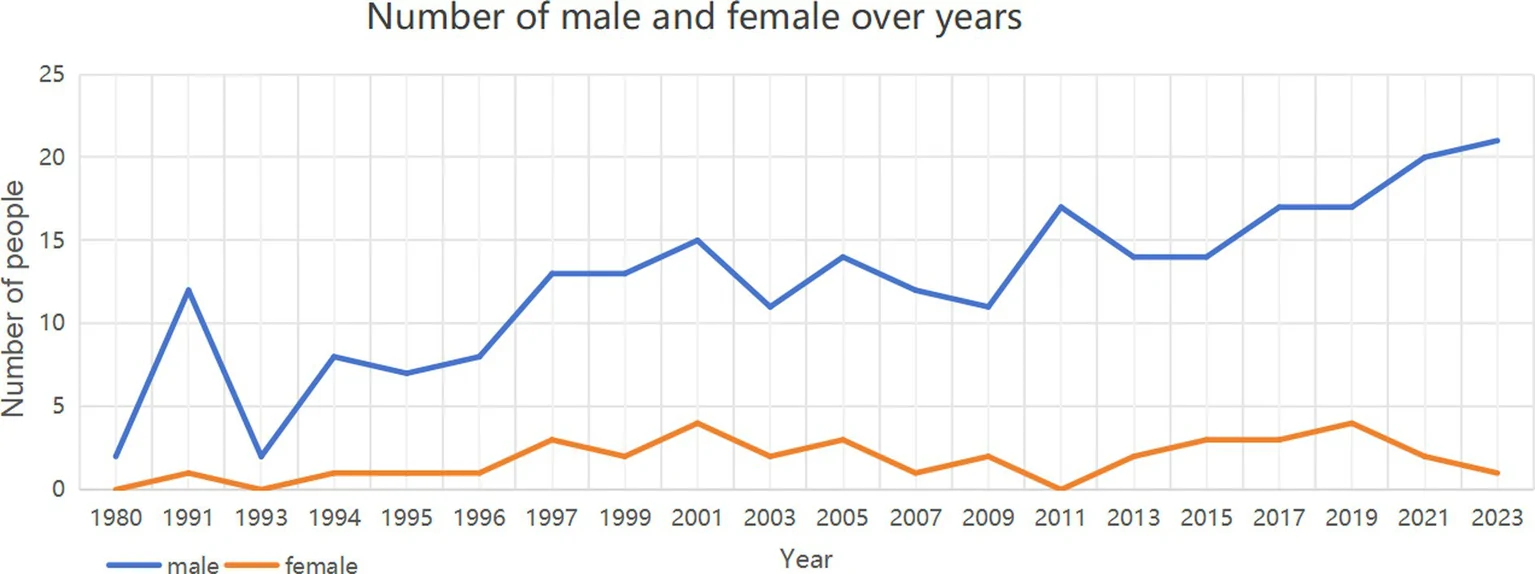
Distribution of male and female biomedical academicians elected to the two academies, 1980–2024.

### 3.3 Cross-group comparison of election age by academy and gender

The independent samples t-test showed that the average election age of CAS academicians was significantly lower than that of CAE academicians (*p* < 0.001). A significant difference was also found between male academicians of the two academies (*p* < 0.001). No significant difference was observed between male and female academicians within the same academy (*p* > 0.05) (Table 1). The average age at election of male academicians in the two academies was (57.52 ± 7.27) years, and that of female academicians was (58.39 ± 6.58) years, with *p* = 0.50 (95% CI −3.40 to 1.65) between groups; the comparison between male and female academicians of the Chinese Academy of Sciences showed *p* = 0.08 (95% CI −6.56 to 0.33); the comparison between male and female academicians of the Chinese Academy of Engineering showed *p* = 0.30, 95% CI −1.60 to 5.13; the difference between the two groups was not statistically significant.

**Table 1:** Comparison of average election age between academicians of the two academies.

Group	Average Election age (years)	Average election age for females (years)	Average election age for males (years)
CAE	59.89 ± 6.54	58.42 ± 6.66	60.11 ± 6.52
CAS	55.69 ± 7.16	58.35 ± 6.69	55.31 ± 7.16
*t*-value	5.12	0.15	5.49
Cohen’s d	0.61	0.01	0.70
95% CI	2.58–5.80	−4.46 to 4.60	3.08–6.53
*p*-value	<0.001	0.98	<0.001

Data are presented as mean ± standard deviation. CAE = Chinese Academy of Engineering; CAS = Chinese Academy of Sciences. Cohen’s *d* represents effect size (0.2 = small, 0.5 = medium, 0.8 = large). The 95% CI is the confidence interval of the mean difference (CAE minus CAS); a CI spanning zero (e.g., female subgroup: −4.46 to 4.60) indicates no statistically significant difference at the 0.05 level.

### 3.4 Geographic distribution of birthplace and upbringing

The geographic distribution of the birthplaces and upbringing regions of biomedical academicians showed significant clustering. A total of 83 individuals (approximately 29% [83/284]) were born in or raised in the Jiangsu-Zhejiang-Shanghai region. Shandong, Hunan, Hubei, and Anhui followed, each with about 15–16 individuals; Sichuan, Fujian, Liaoning, and Beijing each had 10–12 individuals; other provinces accounted for smaller proportions (Figure 2).

**Figure 2:**
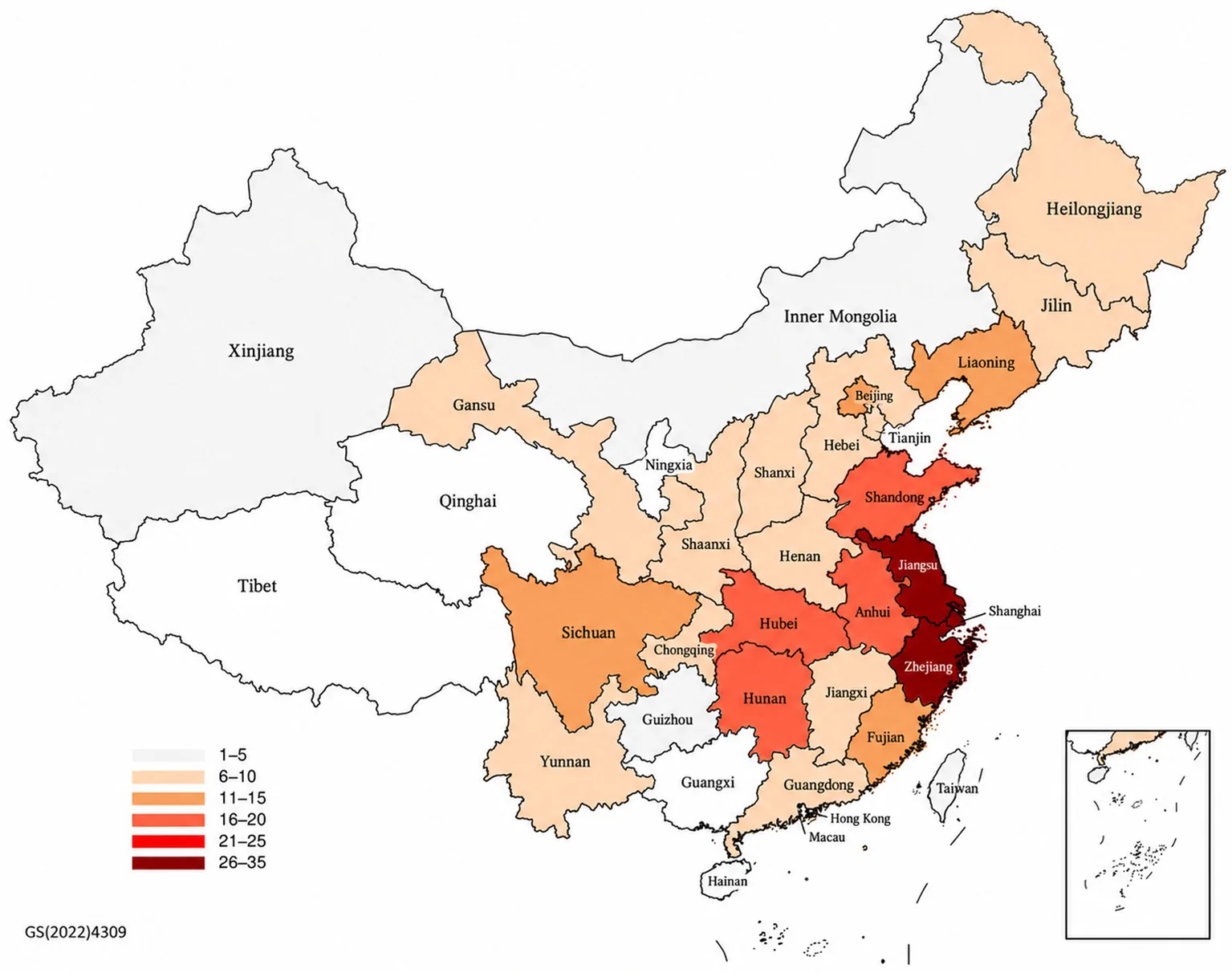
Geographic distribution of birthplaces of biomedical academicians. Deeper shades indicate a larger number of academicians born or raised in that province. The base map was obtained from the Standard Map Service of the Ministry of Natural Resources of China, with no alteration to boundaries. Source: Standard Map Service System of the Ministry of Natural Resources of China (http://bzdt.ch.mnr.gov.cn), review number: GS(2022)4309.

### 3.5 Educational background and overseas training experience

Undergraduate training was concentrated in elite domestic universities: 64.08% (182/284) of academicians completed undergraduate studies at Project 985/211 universities, 34.15% (97/284) from other domestic institutions, and only 1.76% (5/284) received their first degree abroad. At the doctoral level, 33.80% (96/284) held overseas doctoral degrees. The proportion of overseas educational experience increased significantly from the undergraduate to the doctoral stage. Academicians with overseas doctoral degrees were elected at a significantly younger age (*p* < 0.001) (Table 2).

**Table 2:** Comparison of election age between academicians with and without overseas doctoral degrees.

Group	Number	Election Age	Cohen’s *d*	*P*-value	*95%CI*
Overseas Doctoral Degree	96	54.74 ± 7.14	0.63	<0.001	−2.63 to −6.02
No Overseas Doctoral Degree	188	59.07 ± 6.74			

Data are expressed as mean ± standard deviation. *p* values were calculated by the independent-samples *t* test. Cohen’s d indicates effect size (0.2 = small, 0.5 = medium, 0.8 = large). The 95% CI is the confidence interval of the mean difference between the two groups (overseas Doctoral Degree group minus No overseas Doctoral Degree group).

### 3.6 Mentor effect and election age

Among the academicians, 81 (28.52%) were supervised by academician mentors. The independent samples *t*-test showed that academicians with an academician supervisor were elected at a significantly younger age (*p* < 0.05) (Table 3).

**Table 3:** Comparison of election age between those with and without an academician mentor.

Group	Number	Election Age	*t*-value	Cohen’s *d*	*95%CI*	*p*-value
Academician Supervisor	81	56.19 ± 6.66	−2.24	−0.29	−3.86 to −0.17	0.03
No Academician Supervisor	203	58.20 ± 7.31				

Data are presented as mean ± standard deviation. Cohen’s d represents the effect size (0.2 = small, 0.5 = medium, 0.8 = large).

### 3.7 Distribution of employing institutions and disciplines

According to the statistics based on the first listed institution in the official records of academicians, 51.06% of academicians (145/284) were primarily employed by universities, 31.34% (89/284) by independent research institutes, 13.38% (38/284) by clinical hospitals, and 4.23% (12/284) by government health and research management agencies. Some academicians held concurrent positions.

The distribution of disciplines is based on the “National Standard for Discipline Classification and Codes of the People’s Republic of China.” The first-level disciplines involved in the biomedical field include clinical medicine, basic medical sciences, pharmacy, traditional Chinese medicine and Chinese materia medica, preventive medicine and public health, military medicine and special medicine, and biology etc. (20). The main research areas of 284 academicians in the biomedical field were classified according to their first-level disciplines. When an academician’s work spanned multiple fields, the primary discipline was determined based on the academician’s primary departmental affiliation and scholarly output. There were 122 academicians in Basic Medical Sciences (42.96%, 122/284), followed by 58 in Clinical Medicine (20.42%, 58/284), 55 in Biology (19.36%, 55/284), 29 in Pharmacy and Chinese Materia Medica (10.21%, 29/284), 10 in Preventive Medicine and Public Health (3.52%, 10/284), and 10 in Military Medicine and Special Medicine (3.52%, 10/284) (see Appendix A).

### 3.8 Differences in educational level across age groups

All academicians were divided into three groups based on election age: <50 years (younger group), 50–65 years (middle group), and >65 years (older group). Educational level was an ordinal categorical variable. The Kruskal-Wallis H test was used to compare the distribution of educational levels among academicians in different age-at-election groups, and the Dunn-Bonferroni method was subsequently used for multiple pairwise comparisons with significance correction. The results showed that the mean ranks of educational level samples in the three groups were 104.91 (younger group), 135.04 (middle-aged group), and 213.89 (older group), respectively; the overall between-group comparison was *H* = 57.42, *df* = 2, *p* < 0.001; all pairwise comparisons were statistically significant: younger age group vs. middle age group (adjusted *p* = 0.038), younger age group vs. older age group (adjusted *p* < 0.001), middle age group vs. older age group (adjusted *p* < 0.001) (Table 4).

**Table 4:** Comparison of highest educational attainment among biomedical academicians of different age groups.

Group	Test statistic	Std. error	Std. test statistic	*p*-value	Adjusted *p*-value
Younger - Middle	−30.14	12.30	−2.45	0.014	0.038
Younger - Older	−108.99	15.63	−6.97	<0.001	<0.001
Middle - Older	−78.85	11.79	−6.69	<0.001	<0.001

Pairwise multiple comparisons of the Kruskal–Wallis H test (Dunn-Bonferroni method). The test statistic refers to the difference in mean ranks between two groups. Adjusted *p*-value indicates significance after multiple-comparison correction. A two-sided *p* < 0.05 was considered statistically significant.

### 3.9 Multiple regression analysis of factors associated with age at election

We fitted a multiple linear regression model using age at election as the dependent variable; independent variables included gender, highest academic degree, overseas doctoral degree, prestige of the undergraduate institution, academician supervisor status, and type of employing institution. The overall model was statistically significant (*F* = 12.58, *p* < 0.001), with *R*^2^ = 0.21 and adjusted *R*^2^ = 0.19. All VIF values were below 1.3 (range: 1.02–1.29), and the maximum condition index was only 6.03, indicating no multicollinearity. The maximum Cook’s distance was 0.07, well below the conventional threshold of 1, suggesting no influential outliers and confirming model stability.

Regression results showed that highest degree attainment was the strongest and most consistent predictor of age at election. After adjusting for all other variables, each one-unit increase in the degree code (indicating a lower qualification level, moving from doctoral to master’s then bachelor’s) was associated with an average delay of approximately 3.3 years in election age (*B* = 3.256, *p* < 0.001). Having an academician supervisor was independently associated with earlier election, reducing the expected age at election by roughly 1.8 years on average (*B* = −1.840, *p* = 0.033). Gender, prestige of undergraduate institution, overseas doctoral degree, and institutional type showed no independent statistical association after full adjustment (*p* > 0.05; Table 5). Notably, overseas doctoral degree was significantly associated with younger election age in the independent-samples *t*-test analysis. However, after entering the multiple regression model and adjusting for confounders including highest degree, this association lost statistical significance (β = −1.649, *p* = 0.071). This finding suggests the crude association between overseas doctoral degree and earlier election is largely confounded by differences in degree attainment, and higher academic qualifications themselves predict earlier election.

**Table 5:** Multiple linear regression analysis of factors influencing the age at election of biomedical academicians.

Variable	Unstandardized coefficient B	Standard error	Standardized coefficient Beta	*t* -value	*p* -value	VIF
constant	53.091	0.974	—	54.584	<0.001	—
Gender (Male = 0 Female = 1)	0.294	1.157	0.014	0.254	0.800	1.018
First academic degree (0 = Project 985、211 Universities and Overseas Universities, 1 = Regular Colleges and Universities)	−0.375	0.845	−0.025	−0.444	0.657	1.102
Highest academic degree (0 = Doctor Degree, 1 = Master’s, 2 = Bachelor’s)	3.256	0.510	0.386	6.380	< 0.001	1.290
Overseas doctoral degree (0 = No, 1 = Yes)	−1.649	0.910	−0.109	−1.813	0.071	1.270
Academician supervisor (0 = No, 1 = Yes)	−1.840	0.861	−0.116	−2.137	0.033	1.038
Employer (0 = the Academy of Sciences and Research institutions; 1 = universities; 2 = Hospitals; 3 = Government departments, Academic societies, etc.)	0.350	0.511	0.038	0.685	0.494	1.088

Dependent variable = age at election. Coding rules for each categorical predictor are given within variable labels. Negative *B* values indicate predictors associated with younger-age-at-election; positive *B* values correspond to predictors associated with older-age-at-election.

## 4 Discussion

### 4.1 Core characteristics and formation logic of structural imbalance in the biomedical academician community

Drawing on large-sample cross-sectional quantitative analysis, this study identifies multidimensional structural imbalances within China’s top biomedical talent system. It should be emphasized that this is an observational study. The causal interpretations presented below are reasonable inferences grounded in existing literature rather than causal mechanisms directly validated by our data.

(1) The gender distribution persists in imbalance, and the career advancement barriers for high-achieving women remain substantial. Women account for only 12.68% of academicians (members) in the Chinese biomedical sciences, with no significant improvement observed over the past decade, which is consistent with the global “leaky pipeline effect” for women in the medical field (21). In contrast, the National Academy of Medicine (NAM) of the United States has fostered member diversity through adaptive choice architecture (a policy design that nudges equitable selection) since 2008, with women accounting for 50% of newly elected members in 2022 and 40% in 2023 (22). Existing studies show that female medical academics face more complex career barriers than male peers, particularly in accessing high-quality mentorship and career opportunities (23). Social support systems encompassing family, peers, and mentors strongly shape their career progression (24). Although women now make up more than half of medical school entrants globally, their representation shrinks substantially at senior academic ranks. In surgery, for example, despite rising numbers of female medical students, female representation at the professor level remains as low as 3.4% (25, 26).

The critical window for academic promotion and academician selection in medicine falls between ages 40 and 55, overlapping with the peak period of childbearing and family care responsibilities for women, which may interrupt research careers. In addition, high-end academic resources such as research networks, principal investigator roles on major projects, and leadership positions in academic societies carry implicit gender bias. Female researchers consequently have less access to elite mentorship and high-impact academic platforms. These persistent hidden barriers within academic medicine ultimately create severe gender imbalance at the highest academic level. Such cross-national comparison should be interpreted cautiously given substantial differences in nomination and election systems between China and the United States.

(2) Regional resource concentration and the Matthew effect in talent development. The Matthew effect (cumulative advantage) is evident in the strong geographic clustering of birth and formative regions among biomedical academicians, with Jiangsu-Zhejiang-Shanghai holding a clear advantage while central and western regions face shortages of top talent. Research suggests that socioeconomic disparities create unequal access to high-quality basic education, affecting the long-term career development of biomedical talents (27). From the perspective of regional development and WHO’s principle of equitable health workforce distribution, this phenomenon is not a natural outcome of regional economic differences but arises from long-standing inequalities in basic education quality, research platform distribution, research funding allocation, and talent incentive policies.

Economically developed eastern regions offer high-quality basic education, host nearly 30% of China’s national key biomedical laboratories and numerous high-level tertiary hospitals, and maintain mature talent incentive systems and industry-university-research integration frameworks, forming a supportive ecosystem for cultivating top talents. In contrast, central and western regions continue to suffer from resource constraints and weaker talent nurturing conditions. These regional disparities reinforce one another over time, generating a Matthew effect.

(3) Unequal access to training resources: differences in doctoral education and academician mentorship High-quality mentorship increases academic output, leadership capacity, and prospects for academic promotion (28, 29). Univariate analysis indicated that overseas doctoral degree and academician mentorship were both associated with shorter career progression timelines. Multiple regression further confirmed the independent role of academician mentorship, while overseas doctoral degree lost significance after adjustment for degree attainment. These two high-value developmental resources (academician mentorship and international training opportunities) are concentrated in elite universities and eastern developed regions, and remain less accessible to researchers and early-career scholars in central and western areas. Uneven distribution of high-quality training resources may generate disparities at the early talent development stage and ultimately consolidate structural imbalance within the pool of top talents.

In addition, academicians elected before age 50 and those elected between 50 and 65 had significantly higher educational attainment than those elected after age 65. This points to a strong association between age at election and educational capital. The younger cohorts received systematic, high-quality medical training during their formative career stages, enabling substantial accumulation of human capital, laying a solid foundation for high-impact research and major scientific achievements, and conferring advantages in academician selection. Older cohorts faced era-specific constraints, with fewer opportunities to complete master’s and doctoral training early in their careers. This suggests that systematic advanced academic training at an early career stage is a cornerstone for contemporary scholars to achieve major academic breakthroughs (30).

(4) Misalignment between disciplinary composition and practical health needs: the talent structure is heavy on basic sciences, light on clinical medicine, and weak on public health China’s biomedical academician cohort is dominated by basic medical scientists, who account for approximately 42.94% of the total sample, while the share of top talents directly engaged in clinical care and public health prevention and control remains relatively low. By comparison, roughly 30% of members of the U. S. National Academy of Medicine are basic scientists and 63% are clinical practitioners (22). This distribution differs markedly from mature international medical talent structures and deviates from the Healthy China strategic goal of coordinated development between clinical and basic sciences.

Potential contributing factors include the weighting of basic science achievements in the two academies’ selection criteria, and the core mission of hospitals centered on clinical service delivery, leaving limited capacity for sustained long-term basic research. Hospital-based research tends to focus on clinical applications and yields fewer landmark original discoveries; research funding and protected academic time are also less abundant than in universities and dedicated research institutes (31).

Overall, the structural imbalance of top biomedical talents in China is a systemic problem across the entire talent pipeline: from early training and career development to final selection. In recent years, the Chinese government has advanced reforms of the academician system, emphasizing the honorary and academic nature of the title, optimizing selection mechanisms, and adjusting disciplinary composition. These reforms constitute an important institutional response to this systemic imbalance.

### 4.2 Policy implications for the governance of leading medical talents based on the WHO Global Health workforce governance framework

This study identifies structural imbalances in China’s biomedical academician cohort across gender, geography, discipline, and career trajectories. Drawing on global health workforce governance frameworks including the *WHO 2026 National Health Workforce Accounts Report* and the *2030 Accelerating Global Health and Care Workforce Action*, together with China’s Healthy China and talent-strengthening national strategies, we propose policy implications for leading medical talent governance at three levels: national top-level governance, institutional platform empowerment, and autonomous development of young talents.

(1) National top-level governance: addressing dual gender and geographic imbalances. This study finds that only 12.68% of biomedical academicians are female, and this share has not increased among newly elected academicians over the past decade; three election cycles returned no female academicians. Birthplaces and regions where academicians received education and professional training also show strong geographic clustering. Accordingly, it is recommended that gender balance should be weighted more heavily in academician nomination and evaluation within major national talent programs. Targeted support mechanisms and appropriate inclusive measures for outstanding female medical researchers could be explored. At the same time, it may be considered to alleviate the geographical concentration of top medical talents by deploying national research platforms to the central and western regions and implementing a regional talent revitalization plan.
(2) Institutional platform construction: optimizing disciplinary and institutional structures In terms of disciplinary distribution, basic medical scientists account for 42.94% of academicians while clinical medicine accounts for only 20.42%, revealing a marked imbalance between basic and clinical talent. For institutional affiliation, 51.06% of academicians are primarily based in universities, while merely 13.38% work in clinical hospitals. We recommend strengthening research hospitals and clinical research centers and promoting interdisciplinary training bridging clinical medicine and basic medical sciences. Meanwhile, national talent programs could appropriately increase the share of applied leading talents in clinical medicine and public health, and clinical institutions should be given a stronger role in cultivating leading medical talents.
(3) Youth talent development: strengthening mentorship systems and diversifying international training pathways. Multiple regression shows that higher degree attainment predicts election roughly 3.3 years earlier on average, and academician mentorship predicts election approximately 1.8 years earlier. Overseas doctoral degree, by contrast, shows no independent association with age at election after adjusting for confounders. We therefore recommend expanding early-stage research support programs for young medical talents and establishing standardized mentorship matching and training systems. While continuing to support international joint training and overseas doctoral education, priority should also be placed on improving the quality of domestic doctoral training, so overseas doctoral degree does not become a new source of inequality. This will foster a diverse, equitable, and sustainable developmental ecosystem for young leading talents.

### 4.3 Research limitations

This study has several limitations. First, as a retrospective cross-sectional observational study, it can only identify statistical associations and cannot establish causal relationships between specific factors and talent outcomes. Second, the sample is restricted to successfully elected biomedical academicians from the two academies, an elite cohort subject to inherent survivor bias. Findings cannot be generalized to medical researchers with similar educational and mentorship backgrounds who were not elected academicians, nor can they directly answer what determines academic success. Third, this study relies only on publicly available resume-based variables and omits potential confounders such as research funding, team resources, family support, and social capital. Bibliometric indicators (publication output, citations, h-index) were not included, limiting our ability to fully capture the complex drivers of talent development. Fourth, the study does not account for cohort effects related to election year. Changes in academician selection criteria, international mobility policies, and domestic doctoral education systems over time may confound associations between educational experience and age at election. Future research may incorporate election year or adopt cohort-adjusted designs. Fifth, this research focuses on China’s unique dual-academy system, which has country-specific talent selection mechanisms and research funding models; findings should be extrapolated to other national contexts with caution. Future work may adopt longitudinal cohort studies of young biomedical scholars to validate dynamic effects of education and mentorship, and conduct quasi-experimental policy evaluations to quantify the impacts of international training and inclusive talent policies. Expanding the sample to include provincial-level leading medical talents can further enrich evidence for balanced health workforce development.

### 4.4 International research value and generalizability

This study is situated within the context of medical talent development in a large middle-income country, systematically examines the structural imbalance of the high-end health workforce, and provides large-sample empirical evidence from China for global research on leading medical talents. Currently, quantitative cohort studies on Chinese biomedical academicians remain scarce. This article draws on a sample of 284 academicians to depict group characteristics and career growth patterns, filling the gap in research on the structure of top medical talents in middle-income countries, and providing reference data for international comparative studies.

Aligning with the WHO global health workforce governance agenda, this study embeds the analysis of academician cohort imbalance within an international health human resources governance framework, bridging local empirical evidence and global governance standards. It directly addresses WHO’s core concerns over persistent inequalities in the gender, geographic, and skill composition of health workforces, offering empirical support for cross-national comparison and policy design, and Chinese experience for advancing balanced global health workforce development. Against WHO’s projection of an 11-million global health worker shortage by 2030, the gender barriers, regional talent clustering, and disciplinary structural imbalances identified in this study are issues common to many countries globally. Most low- and middle-income countries globally grapple with challenges such as uneven regional distribution of high-level health workforce, insufficient capacity in training top-tier talent, disciplinary structures that are misaligned, and gender inequality in career development. The talent imbalances and resource allocation patterns documented in this paper can provide methodological insights for other countries in conducting cohort studies on medical leadership talent and reforming health workforce governance systems. Furthermore, the multi-layered governance pathway spanning state, institution, and individual proposed in this paper may inform efforts within China and other countries to build inclusive and sustainable medical talent ecosystems.

## 5 Conclusion

Based on multidimensional quantitative analysis of 284 biomedical academicians from CAS and CAE, this study identifies prominent structural imbalances within China’s top biomedical talent system across gender, geography, discipline, and career trajectories. These imbalances manifest as persistently low representation of female leading researchers, heavy geographic concentration of academicians’ origins in eastern regions, uneven disciplinary distribution across basic medical sciences, clinical medicine and public health, and independent positive associations between higher educational attainment, academician mentorship and earlier election. Drawing on the WHO Global Health Workforce Governance Framework and China’s national health development priorities, this paper proposes targeted governance strategies at national, institutional, and individual levels. The findings provide policy implications for reforming China’s national training system for leading medical talents and advancing balanced health workforce governance, as well as broader references for strengthening health workforce systems in other middle-income countries.

## Data Availability

The original contributions presented in the study are included in the article/Supplementary material, further inquiries can be directed to the corresponding author.

## Appendix A

**Table A:** Descriptive statistics of the full sample of academicians in the biomedical field (*N* = 284).

Variable	Category/Statistic	Value
Age at election (years)	Mean ± Standard Deviation	57.63 ± 7.18
Age group, *n*(%)	Young group (<50 years old)	37 (13.02)
	Middle-aged group (50–65 years old)	208(73.23)
	Elderly group (>65 years old)	39 (13.73)
Gender, *n*(%)	Male	248 (87.32)
	Female	36 (12.68)
Undergraduate institution, *n*(%)	985/211 Universities	182 (64.08)
	Foreign institutions	5 (1.76)
	Regular universities	97 (34.15)
Highest degree, *n*(%)	Doctor	186 (65.49)
	Master	30 (10.56)
	Bachelor	68 (23.94)
Overseas doctoral degree, *n*(%)	Yes	96 (33.80)
	No	188 (66.20)
Academician supervisor, *n*(%)	Yes	81 (28.52)
	No	203 (71.48)
Affiliated institution type, *n*(%)	the Academy of Sciences and Research institutions	89 (31.34)
	Universities	145 (51.06)
	Hospitals	38 (13.38)
	Government departments, Academic societies, etc.	12 (4.23)
